# Preservation of long-term memory in older adults using a spaced learning paradigm

**DOI:** 10.1007/s10433-023-00750-5

**Published:** 2023-02-01

**Authors:** Michelle Caffrey, Sean Commins

**Affiliations:** Department of Psychology, Maynooth University, Co. Kildare, Ireland

**Keywords:** Spaced learning, Distributed practice, Massed learning, Long-term memory, Retention

## Abstract

How much information we retain depends on type/schedule of training. It has been widely acknowledged that spaced learning is advantageous compared to massed learning for cognitively healthy young adults and should be considered an educational standard. Literature would suggest that the spacing effect is preserved with age, though it is unclear whether this effect translates to more ecologically valid concepts such as face-name associations, which are particularly susceptible to deterioration with age. Two experiments were conducted to investigate the effects of spacing across recent/remote retention intervals, and the effect of age on spacing in cognitively healthy older adults using the Face-Name Pairs task. Experiment 1 results suggest that the beneficial memory effects of spacing are particularly observed with long-term memory. Experiment 2 results suggest that older adults are impaired at learning compared to younger adults, that the spacing effect influences both older and younger adults at longer intervals, and that spaced-trained participants display similar forgetting patterns at longer intervals, irrespective of age. These results may have some implications regarding improving the conditions under which optimum retention occurs (namely, whether spacing is beneficial when learning ecologically valid concepts at longer intervals outside of laboratory settings), and may provide insight into the effect of age on our ability to learn and remember face-name associations.

## Introduction

It has been widely acknowledged that spaced learning (repeated learning sessions separated by intervals) holds a distinct advantage over massed learning (learning occurs in one sitting), even when overall encoding time does not differ (Benjamin and Tullis 2010; Kapler et al. 2015; Delaney et al. 2018). The spacing effect has been widely reproduced across many domains (Goverover et al. 2009; Wang et al. 2017). For example, Kapler, et al. (2015) found that undergraduate students who reviewed lecture material after an interval of multiple days performed better on tests than those who reviewed the content after only one day. Similarly, research has shown that spaced learning also benefits the retention of practical skills at two-weekly and one-yearly intervals in surgical trainees (Spruit et al. 2014). The findings are so robust that Kapler et al. (2015) have suggested that repeated sessions should be considered as an educational standard. Despite this, individuals consistently rely on massed schedules of learning over spaced, even going so far as to formally judge massed learning as better than spaced when presented with alternative evidence (Kornell and Bjork 2007; Kornell 2009; Son and Kornell 2009). This may be due to the fact that massed learning can be less time-consuming than spaced (Baddeley and Longman 1978).

Encoding is an active and constructive process; memories are not perfect portrayals of events, rather they are a combination of new sensory information and our existing knowledge and world views. As a result, successful encoding is often heavily dependent on both existing knowledge and our ability to draw associations between that knowledge and new information (Brown and Craik 2000). There is significant research to suggest that the medial temporal lobe, specifically the hippocampus, is imperative in the formation of associative memories (Suzuki 2007; Gould and Davis 2008). Research demonstrates that spacing is particularly advantageous when engaging in associative learning (Richter and Gast 2017; Wang et al. 2020; Nakata and Elgort 2021; Penaloza et al. 2022). Among the most commonly used stimuli in spacing research are simple word or image/character pairs (Cepeda et al. 2009; Kupper-Tetzel 2015; Richter and Gast 2017; Wang et al. 2020). Associative learning may be the key as to why spacing is so advantageous.

Three fundamental theories have been proposed to explain the benefits of spacing: deficient processing, study-phase retrieval, and encoding variability. Deficient processing assumes that information is processed and encoded differently under spaced schedules of learning. Due to the lag between spaced study sessions, individuals experience a reduced sense of familiarity with to-be-learned material compared to massed study sessions. As a result, spaced-trained individuals are more likely to engage in deeper processing and encoding which allows for a stronger long-term memory trace (Hintzman 1974; Limons and Shea 1988; Benjamin and Tullis 2010; Delaney et al. 2010; Maddox 2016). Study-phase retrieval refers to the likelihood of subsequent study sessions separated by time prompting the retrieval of initial study sessions, thus strengthening a memory through constant retrieval that is not present in massed training schedules (Thios and D’Agostino 1976; Benjamin and Tullis 2010; Maddox 2016). Encoding variability theory suggests that an increase in time between study sessions allows for greater variability in encoding. This can refer to a number of factors, such as encoding strategy, context, and the possibility of each study session leaving a distinct and individual memory trace (Bray et al. 1976; Glenberg 1979; Benjamin and Tullis 2010). The key component of encoding variability theory is that individuals are more likely to form strong associations between target information and various contextual cues, thus enhancing retrieval. Combining the aforementioned theories suggests that spaced learning is superior due to more efficient processing and creation of a stronger long-term memory trace upon successful retrieval, as retrieval becomes more difficult when study sessions are spaced apart. Additionally, spaced study sessions allow for the encoding of greater contextual cues which may be associated with target information, thus allowing for a stronger overall memory trace, particularly at longer intervals.

Despite the robustness of the effect, spacing research has been criticized, with some suggesting that laboratory-based tasks are too simple and therefore not indicative of the complex cognitive abilities required in real-world settings (Hochhalter et al. 2005; Logan and Balota 2005; Rohrer and Pashler 2010; Kapler et al. 2015). This raises questions about whether spacing may be of benefit when learning more ecologically valid concepts, such as face-name associations. Reason and Lucas (1984) and Cohen and Faulkner (1986) demonstrated that individuals find it more difficult to recall names than occupations or hobbies and that retrieval blocks are more common with regard to names than any other words. Cohen (1990) concluded that in general, names are only well-remembered when they have meaning; names that lack personal significance are inconsequential and often, individuals have nothing or no one with whom they may be associated, thus making them harder to recall then other semantic concepts. Carpenter and DeLosh (2005) found that participants were better at recalling face-name pairs following the utilization of a spaced, tested schedule when compared to those in the massed condition, suggesting that spacing is beneficial when learning face-name associations.

Furthermore, though spacing has been demonstrated at longer intervals (Price Kerfoot et al. 2010; Spruit et al. 2014), there has been somewhat limited research with regard to the long-term effects of spaced versus massed training schedules when learning more ecologically valid concepts, with many studies performing retests within a week of learning. Simanton and Hansen (2012) evaluated the ability of medical students to retain relevant knowledge across four years depending on the use of different educational models. Their results suggest that clinical application and spaced training schedules may lead to better retention of medical knowledge over a four-year period. These results complement those of Spruit et al. (2014). Similarly, Price Kerfoot et al. (2010) divided urology residents into online spaced training and web-based teaching (massed) schedules, whereby students received information to be studied at scheduled daily intervals, or all together in one single email. Participants were then tested periodically over a forty-five-week period. Results indicated that although participants in the massed condition tended to perform better in the short-term (weeks fourteen to sixteen), participants in the spaced condition demonstrated significantly better long-term retention of material (weeks eighteen to forty-five). These findings suggest that spacing may not be particularly beneficial in the short-term but can lead to significant long-term retention over greater periods of time (Price Kerfoot et al. 2010). This could also explain why many individuals believe that massed learning is preferable to spaced.

Across two experiments, we set out to extend the current knowledge with respect to spacing effects across short- and long-term recall intervals and across different age cohorts. We have chosen to use a face-name association task as this task has been shown to be impacted by age (Martschuk and Sporer 2018)—the task is also known to be hippocampal-dependent (a brain region particularly vulnerable to old age and age-related diseases) (Smith et al. 2014). The face-name pairs task is also more abstract and ecologically valid compared to other commonly used learning tests. In experiment 1, we examine face-name retention at 24 h, 1 week, and 1 month in young adults that have been either spaced- or massed-trained. We hypothesize that spaced learning will preserve memory primarily at longer intervals. In experiment 2, we examine recall of face-name pairs at 24 h and 1 month in a cohort of younger and older adults that have been either spaced- or massed-trained. We hypothesize that younger adults will learn and recall more information generally but that spacing benefits will be observed in both age cohorts.

## Experiment 1

### Methods

#### Participants

A priori power calculations were done to estimate the number of participants required to determine a main effect of spacing. Using fixed effects ANOVAs and an effect size of 0.3 (see Strickland-Hughes et al. 2020) with power of 0.9, *p* = 0.05, and 6 groups (spaced/massed at 24 h/1 week/1 month recall, see below) estimates 118 participants. One-hundred-and-eighteen participants (60 males, 58 females) aged 18–25 (mean = 23.08, standard deviation (SD) = 8.501) participated in the experiment. An exclusion and inclusion criteria were used before recruitment, so all participants were healthy, cognitively healthy, and had normal or corrected-to normal vision. No participant had a known history of drug or substance abuse, and no other relevant medical conditions.

#### Materials

Three control tasks were used to ensure that both training conditions (massed & spaced) were similar in terms of IQ, executive functioning, and general memory ability: The National Adult Reading Test (NART; Nelson 1982) gave an estimate of verbal IQ, the Trail Making Tasks (TMT; Reitan and Wolfson 1992) tested executive functioning, and the Rey Auditory Verbal Learning Test (RAVLT; Rey 1941) evaluated memory and learning strategies. A version of the Face-Name Pairs task (similar to that used by Zeineh et al. (2003)) was used to assess associative memory and was carried out using a Sony laptop. Eight female faces with associated names were presented twice in a block. Each face, a black and white photograph and without hair, was presented on screen for 5 s with the accompanying name. There were 4 blocks in total which were either presented sequentially on the same day (massed condition, *n* = 57) or one block of face-name pairs was presented each day for 4 days (spaced condition, *n* = 61). After each block, retention was assessed. Retention consisted of the 8 faces presented once without their corresponding name. The number of correctly recalled names associated with each of the 8 faces (out of 8) was used to measure memory performance.

#### Procedure

**Fig. 1:**
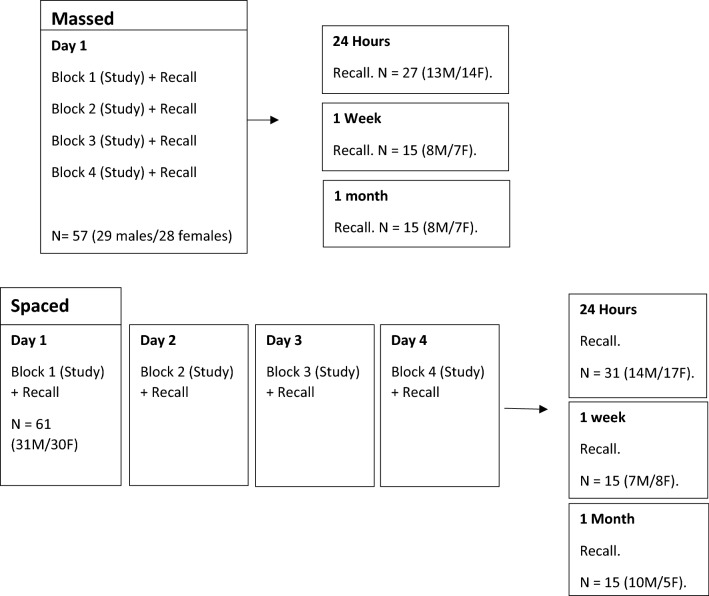
A visual representation of the experimental design and a breakdown of the spaced and massed conditions including the N/interval (experiment 1)

Participants were initially presented with a consent form to be read and signed. The experiment took place in a quiet room, free of distractions. Participants were asked to complete the NART, TMT, and RAVLT prior to partaking in the experiment. Participants were randomly assigned to either the spaced or massed condition and then to a 24 h, 1 week, or 1 month recall condition. Each condition included 4 study blocks and 1 retention block. Participants in the spaced condition completed the 4 blocks over four consecutive days. Participants in the massed condition completed the 4 blocks on one day. Those in the 24 h condition completed a single retention block 24 h after completing the study block, those in the 1 week condition completed the retention block 1 week after completing the study block, and those in the 1 month condition completed the retention block 30 days after the study block (see Fig. 1 for details of conditions, N/condition, and breakdown by gender).

#### Statistics

Microsoft Excel and an IBM SPSS statistics software programme (version 28) were used to calculate the results. Mixed and between factorial ANOVAs were used to compare the learning and recall phases, respectively. Tukey HSD test was used for between group *post-hoc* comparisons and Bonferroni corrected *t*-tests were used for further within-group comparisons. Independent and paired samples *t*-tests were used where appropriate. Results were determined as statistically significant when *p* < 0.05.

#### Ethics

The American Psychological Association and Psychological Society of Ireland codes of ethical conduct were observed throughout. Participants were provided with an information sheet in advance of the experiment, explaining the procedure in detail. All participants were over 18, consented to taking part, and were informed that they could withdraw at any time. Data were anonymized for privacy. All experiments were approved by Maynooth University ethics committee (reference SRESC-2017-097).

### Results

To ensure that both spaced- and massed-trained conditions were matched across age and control tasks, we used a MANOVA to compare participants from both conditions with respect to age and scores on the NART, TMTs, and RAVLT (see Table 1). The results indicate that there was no significant difference between the spaced and massed conditions on the combined dependent variables (*F*(6, 106) = 0.749, *p* = 0.611). There was also no significant difference between conditions when the results were considered separately, suggesting that participants were cognitively-matched and that further results were not affected by these variables.

**Table 1:** Mean age, NART, TMT, and RAVLT scores (standard deviation) for both spaced and massed conditions, and their *p* values

	N	M/F	Age	NART	TMTa	TMTb	TMTb-a	RAVLT
Spaced (SD)	61	31/30	23.59 (9.314)	24.27 (12.466)	25.00 (5.737)	45.88 (15.798)	20.84 (16.015)	52.71 (7.620)
Massed (SD)	57	29/28	22.86 (7.684)	23.47 (12.388)	24.18 (8.892)	46.44 (14.955)	22.30 (13.062)	50.60 (8.088)
*p* values	–	–	0.650	0.735	0.560	0.846	0.596	0.155

To ensure that both spaced- and massed-trained conditions were matched across IQ, we conducted a further MANOVA to compare participants from both conditions with respect to age and predicted full scale, verbal, and performance IQ scores on the NART (see Table 2). The results indicate that there was no significant difference between the spaced and massed conditions on the combined dependent variables (*F*(4, 108) = 1.071, *p* = 0.375). There was also no significant difference between conditions when the results were considered separately, suggesting that participants were IQ-matched and that further results were not affected by these variables.

**Table 2:** Mean predicted full scale, verbal, and performance IQ scores (standard deviation) for both spaced and massed conditions, and their *p* values

	N	M/F	Age	Full Scale IQ	Verbal IQ	Performance IQ
Spaced (SD)	60	31/30	23.59 (9.314)	114.09 (5.564)	112.05 (5.086)	113.09 (4.959)
Massed (SD)	57	29/28	22.86 (7.684)	113.77 (6.182)	111.79 (5.653)	112.77 (5.510)
*p* values	–	–	0.650	0.775	0.795	0.748

#### Acquisition phase

**Fig. 2:**
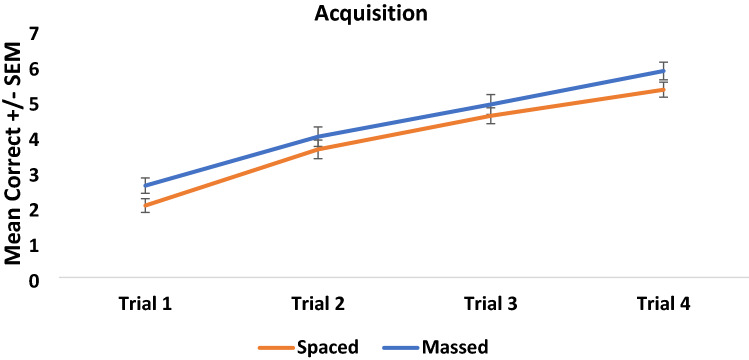
Mean acquisition score (and standard error of the mean, SEM) for both spaced and massed conditions across the four learning blocks

An initial 2 × 4 mixed between-within factorial ANOVA was conducted to compare learning across the 4 trials for both the spaced- and massed-trained conditions. A significant main effect of Trial (*F* (3, 114) = 164.176, *p* < 0.001, partial eta squared = 0.812), but no effect of Condition (*F* (1, 116) = 2.322, *p* = 0.130, partial eta squared = 0.020) was found. There was no significant interaction between trial and condition (*F* (3, 114) = 0.393, *p* = 0.758, partial eta squared = 0.010) (see Fig. 2).

#### Retention phase

**Fig. 3:**
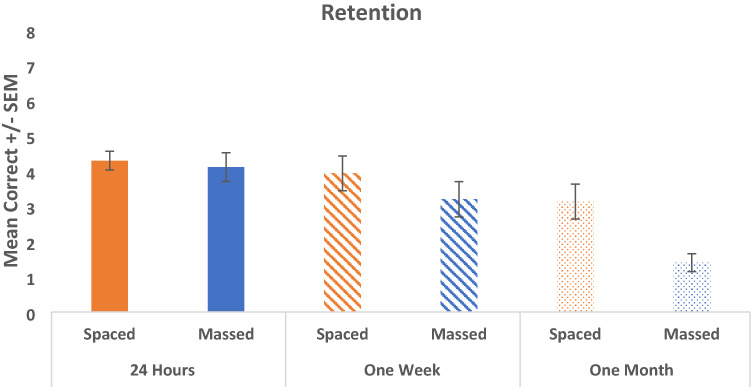
Mean retention score (and standard error of the mean, SEM) for both spaced and massed conditions when retested at 24-h, 1-week, and 1-month post-learning

A two-way between groups ANOVA was conducted to explore the difference between the ability of those in the spaced and massed conditions to recall the face-name pairs following an interval of 24 h, 1 week, or 1 month. The results indicate a main effect of Condition (spaced/massed) (*F* (1, 112) = 9.464, *p* = 0.003, partial eta squared = 0.078) and a significant main effect of Retention Interval (*F* (2, 112) = 14.673, *p* < 0.001, partial eta squared = 0.208). There was no significant interaction effect (*F* (2, 112) = 0.619, *p* = 0.540) (see Fig. 3).

Two one-way ANOVAs were conducted to examine whether there were any differences between the performance of participants in the spaced and massed conditions, respectively, at the three different time intervals. Results for the spaced condition indicated that there was an overall significant difference between performance at each of the three intervals (*F* (2, 58) = 5.110, *p* = 0.009). Tukey HSD *post-hoc* comparisons indicated that performance for the spaced condition was not significantly different at 24 h and 1 week (*p* = 0.183) but there was a significant difference between recall at 24 h and 1 month (*p* = 0.009). *There was no significant difference between performance at 1 week and 1 month* (*p* = 0.505). Similarly, the results of the massed condition indicated that there was a significant difference between performance at each of the three intervals (*F* (2, 54) = 10.482, *p* < 0.001). *Post-hoc* comparisons indicated that performance for the massed condition was significantly different at 24 h and 1 month (*p* < 0.001), and *significantly different at 1 week and 1 month* (*p* = 0.026). There was no significant difference between performance at 24 h and 1 week (*p* = 0.281).

## Brief discussion

These results would suggest that with spaced training there is a small decline in memory performance after 1 week but a limited decline after this. Whereas with massed training, there is a gradual decline in performance throughout the month, and particularly between 1 week and 1 month. The beneficial memory effects of spaced training are particularly observed at long-term intervals (i.e., 1 month). Our results are in line with the original hypotheses and many of the aforementioned studies in that we observed a spacing effect at longer retention intervals, however, according to existing literature we should also expect to observe spacing at shorter intervals which was not the case (Goverover et al. 2009; Benjamin and Tullis 2010; Kapler et al. 2015; Wang et al. 2017; Delaney et al. 2018). This discrepancy may be due to the type of material (face-name associations) or the schedule of spacing implemented. In particular, these results are not dissimilar to those of Price Kerfoot et al. (2010), who found that participants in the massed condition performed better than those in the spaced condition at shorter time intervals, but at longer time intervals, those in the spaced condition retained significantly more information than those in the massed condition. However, it is worth noting that Price Kerfoot et al. (2010) conducted retests at significantly longer intervals in comparison with this experiment (16 and 45 weeks, respectively). Differences in results may be attributable to different types of to-be-learned information (medical knowledge versus face-name associations). Having established that spacing optimizes retention in younger adults using a face-name pairs task, it raises the question of whether the same is true for cognitively healthy older adults, and if so, can spacing be used to help combat natural memory decline with age?

## Experiment 2

### Introduction

Though most studies examining the spacing effect include younger participants, there are a few that focus on cognitively healthy older adults. Bercovitz et al. (2017) concluded that, although younger adults remember more than older adults overall, there is evidence of the spacing effect in both participant groups at 10-day intervals. Similarly, Balota et al. (1989) found that older participants were influenced by the spacing effect, particularly at longer intervals. Therefore, like younger adults, older adults may also benefit from implementing spaced schedules when attempting to learn face-name associations, particularly at longer intervals.

Retention of face-name associations becomes a key difficulty with age (Ozen et al. 2010; Humphries et al. 2015; Hromas and Bauer 2019). For example, D’Argembeau and Van der Linden (2010) found that older adults had more difficulty recalling unfamiliar faces compared to younger adults, while Martschuk and Sporer (2018) noted that younger participants performed better than older participants across a number of different face recognition measures. Age is not always indicative of memory performance. Chalfonte and Johnson (1996) found no difference between the ability of older and younger participants to remember individual objects and colors. However, when asked to recall object/color associations, older adults performed significantly worse than younger adults. Similarly, Grady (2012) acknowledges that while episodic, verbal, and working memory deteriorate with age, semantic memory is largely preserved. Indeed, Naveh-Benjamin et al. (2004) suggest that when it comes to remembering faces and names, older adults particularly struggle with associative memory. Their results indicate that older adults are just as capable as younger adults at remembering names, and exhibit only a slight decline in performance when recalling faces. However, older adults were significantly worse than younger adults when recalling face-name associations. This begs the question of whether spacing may be beneficial to older adults in attempting to recall associative concepts?

Recently, research tends to shy away from encoding variability as an explanation of spacing as it is difficult to control for and thus prove (Benjamin and Tullis 2010; Maddox 2016). However, encoding variability may explain why older adults do not benefit from spacing to the extent of younger adults (Bercovitz et al. 2017). It has long been suggested that natural memory decline with ageing may be a result of an inability to adequately associate information when creating complex memories (Chalfonte and Johnson 1996; Naveh-Benjamin et al. 2000). Furthermore, there is evidence to suggest that older adults are unable to apply context-specific cues in the way that younger adults can, implying that while encoding variability may be beneficial to an extent, older adults cannot make full use of encoded contextual elements (Smith et al. 1998). More recent evidence suggests that older adults are susceptible to hyper-binding, an effect where associations are formed between target information and distractors, which younger adults would be more likely to successfully reject (Powell et al. 2018). In this scenario, encoding variability could work against older adults, allowing for too much association and thus making it difficult to recall the target stimulus. This raises questions about the suitability of spacing as a learning technique for both older and younger adults. There is evidence to suggest that older adults also benefit from spacing (Balota et al. 1989; Bercovitz et al. 2017); however, if the benefits are minimal spacing may not be worth the time and effort required. Experiment 2 will examine this further.

### Methods

#### Participants

A priori power calculations were done to estimate the number of participants required to determine a main effect of spacing and age group and an interaction effect between the two. Using fixed effects ANOVAs and an effect size of 0.3 (see Strickland-Hughes et al. 2020) with power of 0.9, *p* = 0.05, and 8 groups (younger/older, spaced/massed, 24 h/1 month recall, see below) estimates 118 participants. One hundred-and-forty-one participants (67 males and 74 females) were recruited for this study. Based on the recommendation of the World Health Organisation (2015) at the time of data collection, we classified older adults as those aged 55 + . In our sample, older adults were aged 55–87 (mean = 64.63 SD = 9.004). Those classified as younger adults were aged 18–29 (mean = 21.85 years, SD = 2.294). An exclusion and inclusion criteria were used before recruitment, so all participants were healthy, cognitively healthy, and had normal or corrected-to normal vision. No participant had a known history of drug or substance abuse, and no other relevant medical conditions.

#### Materials

Four control tasks were again used to ensure that both conditions (massed and spaced) within each age cohort were similar in terms of IQ, executive functioning, and general memory ability. These tasks included the NART (Nelson 1982), the TMT (Reitan and Wolfson 1992), and the RAVLT (Rey 1941). The Montreal Cognitive Assessment (MoCA; Nasreddine et al. 2005), which tests general cognition and for mild cognitive impairment, was given to the older adults to ensure that all were cognitively healthy. The Face-Name Pairs task used in experiment 1 was used again to assess associative memory.

#### Procedure

All participants were presented with a consent form to be read and signed. The experiment took place in a quiet room, free of distractions. Participants were asked to complete the NART, TMT, RAVLT, and MoCA prior to partaking in the experiment. Each test was explained in full, and results were given upon completion if requested. Similar to experiment 1, participants in each age cohort were randomly assigned to spaced or massed conditions and then to the 24 h or 1 month intervals (see Table 3 for details of N). Each condition again included 4 study blocks and 1 retention trial block. Participants in the spaced condition completed the 4 trial blocks over 4 consecutive days. Participants in the massed condition completed the 4 trial blocks on 1 day. Those in the 24 h condition completed the retention block 24 h after completing the study block and those in the 1 month condition completed the retention block 30 days after the study block. This experiment was also approved by the Maynooth University ethics committee (reference SRESC-2017-097).

**Table 3:** Number of participants in each condition (massed and spaced) and time interval (including gender breakdown)

	24 h	One month
*Spaced*		
Older adults Younger adults	14 (9 female/5 male) 22 (11 female/11 male)	15 (7 female/8 male) 15 (10 female/5 male)
	36	30
*Massed*		
Older adults Younger adults	15 (9 female/6 male) 23 (11 female/12 male)	15 8 female/7 male 22 (12 female/10 male)
	38	37
Total number	74	67

### Results

To ensure that both younger and older spaced- and massed-trained participants were matched across control tasks, we used two MANOVAs to compare participants from both conditions with respect to scores on the NART, TMTs, and RAVLT (see Table 4). The results of the younger MANOVA indicate that there was no significant difference between the spaced and massed conditions on the combined dependent variables (*F*(6, 28) = 1.008, *p* = 0.440). There was also no significant difference between conditions when the results were considered separately. The results of the older MANOVA indicate that there was no significant difference between the spaced and massed conditions on the combined dependent variables (*F*(5, 52) = 1.376, *p* = 0.249) (see Table 4). There was also no significant difference between conditions when the results were considered separately. Again, gender was matched for both age cohorts.

**Table 4:** Mean age, NART, TMT, RAVLT and MoCA scores (standard deviation) for both spaced and massed conditions, and their *p* values

	N	M/F	Age		NART (No errors)		TMTa (S)	TMTb (S)	TMTb-a (S)	RAVLT No correct (Sum 1–5)	MoCA (Score)
Young spaced (SD)	37	21/16		22.32 (2.11)		15.59 (3.362)	32.2 (15.85)	38.4 (13.8)	18.8 (9.55)	48.8 (8.349)	28.6 (0.894)
Young massed (SD)	45	22/23		21.47 (2.39)		13.63 (5.468)	24.83 (7.737)	45.22 (13.25)	20.88 (11.043)	56.73 (12.17)	28.43 (1.455)
*p* values	–	–		–		0.492	0.104	0.297	0.694	0.172	0.807
Older spaced (SD)	29	13/16		65.17 (10.1)		11.36 (6.623)	31.54 (12.55)	81.54 (54.07)	48.68 (46.71)	47.75 (8.077)	27.13 (1.807)
Older massed (SD)	30	13/17		64.1 (7.99)		9.13 (3.646)	32.47 (9.980)	65.45 (13.92)	32.49 (12.955)	47.10 (11.040)	27.57 (1.633)
*p* values	–	–		–		0.116	0.753	0.121	0.073	0.8	0.422

To ensure that both spaced- and massed-trained younger and older adults were matched across IQ, we conducted a further MANOVA to compare participants from both conditions with respect to age and predicted full scale, verbal, and performance IQ scores on the NART (see Table 5). The results of the *younger* MANOVA indicate that there was no significant difference between the spaced and massed conditions on the combined dependent variables (*F*(4, 84) = 0.662, *p* = 0.620). There was also no significant difference between conditions when the results were considered separately, suggesting that participants were IQ-matched and that further results were not affected by these variables. The results of the *older* MANOVA indicate that there was no significant difference between the spaced and massed conditions on the combined dependent variables (*F*(4, 53) = 1.569, *p* = 0.196). There was also no significant difference between conditions when the results were considered separately, suggesting that participants were IQ-matched and that further results were not affected by these variables.

**Table 5:** Mean predicted full scale, verbal, and performance IQ scores (standard deviation) for both younger and older adults in the spaced and massed conditions, and their *p* values

	N	M/F	Age	Full Scale IQ	Verbal IQ	Performance IQ
Young spaced (SD)	37	21/16	22.32 (2.11)	111.41 (5.297)	109.69 (4.928)	110.72 (4.774)
Young massed (SD)	45	22/23	21.47 (2.39)	112.16 (6.816)	110.35 (6.264)	111.35 (6.108)
*p* values	–	–	–	0.591	0.607	0.615
Old spaced (SD)	29	13/16	65.17 (10.1)	116.76 (8.149)	114.59 (7.562)	115.45 (7.129)
Old massed (SD)	30	13/17	64.1 (7.99)	119.24 (4.580)	117.00 (4.234)	117.76 (3.997)
*p* values	–	–	–	0.158	0.139	0.134

#### Acquisition phase

**Fig. 4:**
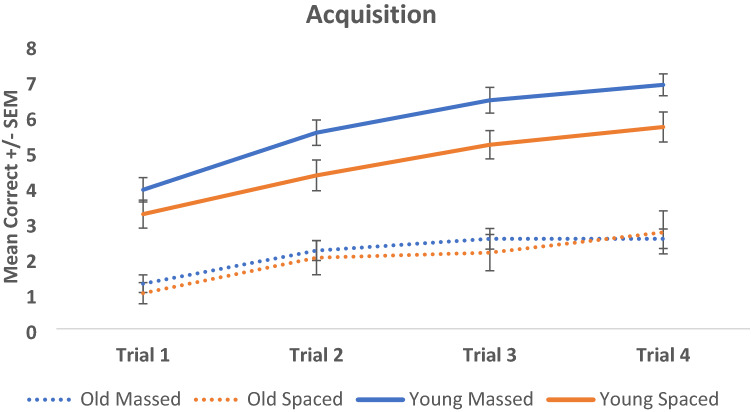
Mean acquisition (and SEM) for both spaced and massed, and older and younger cohorts across the four learning blocks

An initial 4 × 4 mixed between-within factorial ANOVA was conducted to compare learning across the 4 trials for both the spaced- and massed- trained conditions and for both the younger and older cohorts. There was a significant main effect of Trial (*F* (3, 135) = 84.323, *p* < 0.001, partial eta squared = 0.652) and a significant effect of Cohort (older/younger) (*F* (3, 137) = 39.135, *p* < 0.001, partial eta squared = 0.461). There was a significant interaction between Trial and Cohort (*F* (9, 328.705) = 3.019, *p* = 0.002, partial eta squared = 0.062) (see Fig. 4). Bonferroni-corrected t-tests indicate that the mean number of correct responses on trial 4 were significantly higher than trials 1, 2, and 3 (*p* < 0.001), suggesting that all groups learned the task. *Post-hoc* comparisons using the Tukey HSD test indicated that there was an overall significant difference between the performance of the young and old cohorts (*p* < 0.001). There was also a small but significant difference between the performance of the young spaced and massed conditions (*p* = 0.022), but no significant difference between the performance of the old spaced and old massed conditions (*p* = 0.973).

#### Retention

**Fig. 5:**
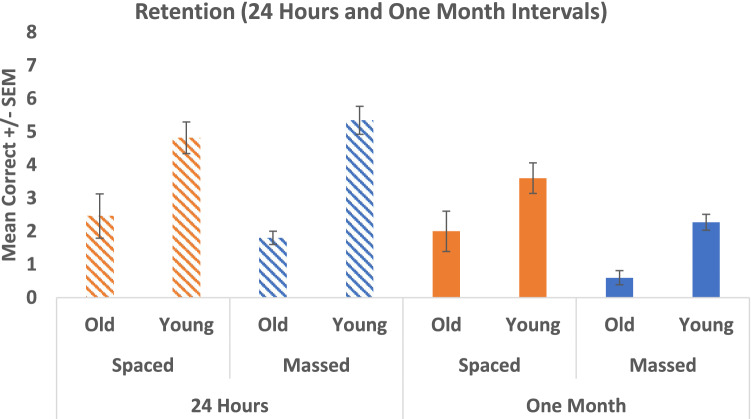
Mean retention scores (and SEM) for both spaced and massed, and older and younger cohorts when retested at 24 h and 1 month

An initial 2 × 2 × 2 factorial ANOVA was conducted to explore the difference between the ability of those in the spaced and massed conditions and the ability of younger and older participants to recall the face-name pairs following an interval of 24 h or 1 month. There was a significant main effect of Time (*F* (1, 132) = 20.246, *p* < 0.001, partial eta squared = 0.133), a significant effect of Age (*F* (1, 132) = 48.087, *p* < 0.001, partial eta squared = 0.267), and an effect of Condition (*F* (1, 132) = 4.667, *p* = 0.033, partial eta squared = 0.034). There was a significant interaction between Age and Time (*F* (1, 132) = 3.954, *p* = 0.049, partial eta squared = 0.029), but no significant interaction between Condition and Age (*F* (1, 132) = 0.912, *p* = 0.341, partial eta squared = 0.007), no significant interaction between Condition and Time (*F* (1, 132) = 3.846, *p* = 0.052, partial eta squared = 0.028), and no significant interaction between Condition, Time, and Age (*F* (1, 132) = 0.714, *p* = 0.400, partial eta squared = 0.005) (see Fig. 5).

To examine the differences in retention in more depth, we carried out two further 2 × 2 factorial ANOVAs. Recall at 24 h indicated that there was a significant main effect of Age (*F* (1, 69) = 37.197, *p* < 0.001, partial eta squared = 0.350), with older adults recalling less names compared to younger adults. However, there was no effect for Condition (*F* (1, 69) = 0.019, *p* = 0.892, partial eta squared < 0.001) and no interaction effect between Age and Condition (*F* (1, 69) = 1.514, *p* = 0.223, partial eta squared = 0.021). Recall at 1 month also showed a significant effect of Age (*F* (1, 63) = 13.373, *p* = 0.001, partial eta squared = 0.175), with older adults again showing poor recall. There was also a significant effect of Condition (*F* (1, 63) = 9.287, *p* = 0.003, partial eta squared = 0.128), with *those in the spaced condition recalling significantly more than those in the massed condition* (irrespective of age). There was no interaction between Age and Condition (*F* (1, 63) = 0.007, *p* = 0.935, partial eta squared < 0.001) (see Fig. 5).

#### Forgetting

**Fig. 6:**
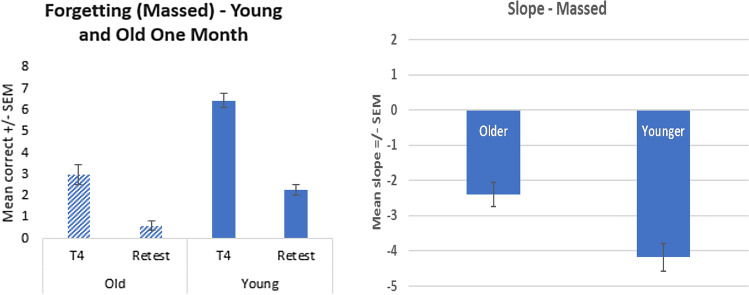
Mean recall scores comparing the final trial of learning to scores recalled 1 month later (left), and the equivalent forgetting slope (right), for the older and younger massed conditions

Our results suggest that participants in the spaced condition (irrespective of age) recalled more compared to those in the massed condition and that this effect was observed at 1 month recall. As such, we would expect a greater forgetting effect (between the final learning trial compared to the recall trial) for the massed condition compared to the spaced condition, particularly at the 1 month recall. Figure 6 shows a large and significant forgetting effect for both the younger (*t* (21) = 9.970, *p* < 0.001) and older cohorts (*t* (14) = 7.159, *p* < 0.001) in the massed condition. Interestingly, the rate of decline is significantly worse for the younger compared to the older adults (mean slope for younger adults is − 4.18 ± 0.4 and for older adults is − 2.4 ± 0.33, *t* (35) = 3.074, *p* = 0.004).

**Fig. 7:**
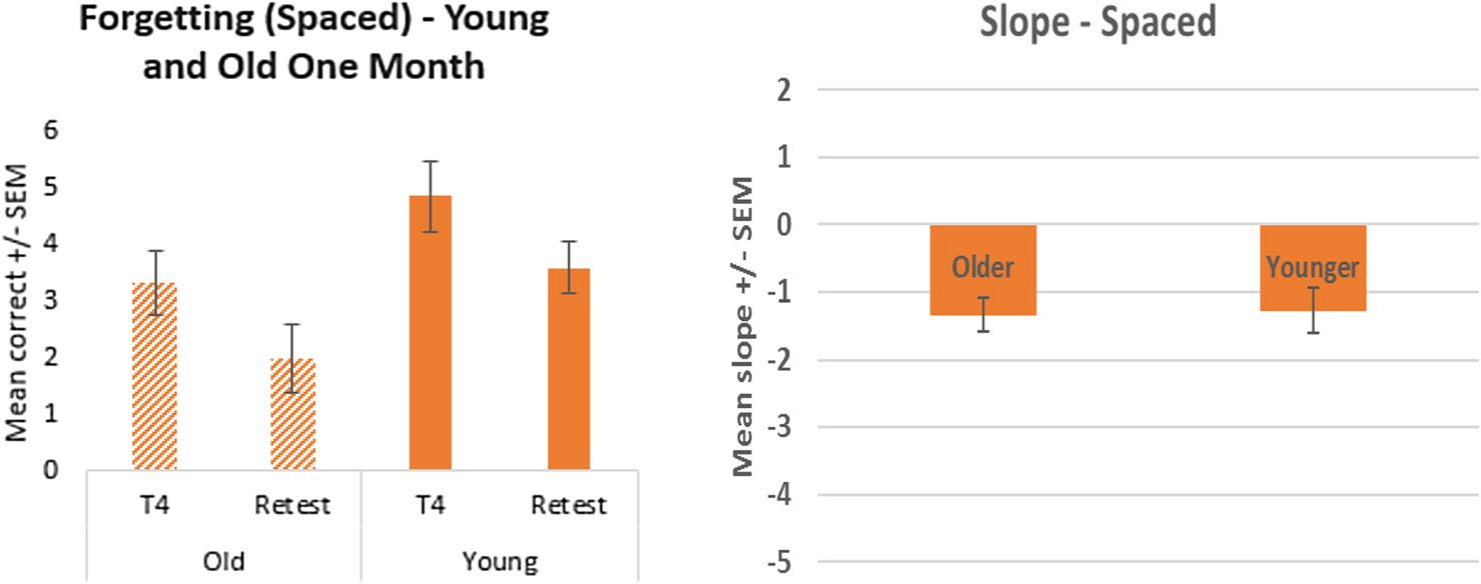
Mean recall scores comparing the final trial of learning to scores recalled 1 month later (left), and the forgetting slope (right), for the old and young spaced cohorts

Figure 7 also shows a significant forgetting effect for both the younger (*t* (14) = 3.67, *p* < 0.001) and older cohorts (*t* (14) = 5.29, *p* < 0.001) in the spaced condition. In contrast to the massed condition, the rate of decline for both age cohorts (mean slope for younger adults is -1.266 ± 0.33 and for older adults is − 1.33 ± 0.25) is similar with no significant difference (*t* (28) =  − 0.156, *p* = 0.877). Overall, the rate of decline is significantly worse for the massed condition compared to the spaced condition (*F* (1,63) = 28.4, *p* < 0.001).

**Fig. 8:**
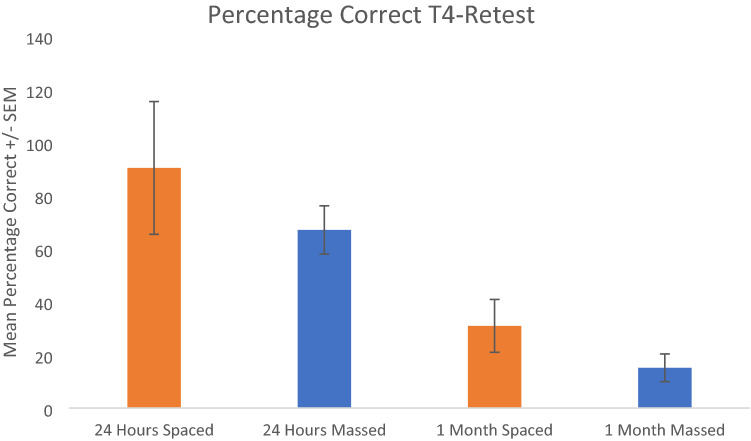
A comparison of the percentage of correctly recalled face-name pairs between Trial 4 and the Retest in older adults at both 24 h and 1 month

A further 2 × 2 factorial ANOVA was conducted to explore the differences between older adults at both intervals in terms of percentage of correctly retained face-name pairs from Trial 4 to the Retest. Results indicated that there was a significant main effect of Interval (*F* (1, 56) = 16.207, *p* < 0.001, partial eta squared = 0.224), with older adults recalling a lower percentage of face-name associations at 1 month. However, there was no effect for Condition (*F* (1, 56) = 1.934, *p* = 0.170, partial eta squared = 0.033) and no interaction effect between Interval and Condition (*F* (1, 56) = 0.057, *p* = 0.813, partial eta squared = 0.001) (see Fig. 8).

## Discussion

Findings from experiment 1 show a strong effect of spaced learning at long-term intervals (1 month). This is somewhat in line with our original hypothesis and other studies; however, we would also have expected to see evidence of spacing at shorter intervals which was not the case (Goverover et al. 2009; Benjamin et al. 2010; Kapler et al. 2015; Wang et al. 2017; Delaney et al. 2018). In particular, these results are somewhat similar to those of Price Kerfoot et al. (2010), who found that participants in the massed condition performed better than those in the spaced condition at shorter time intervals, but at longer time intervals, those in the spaced condition retained significantly more information than those in the massed condition. This finding would suggest that at shorter intervals, the schedule of learning makes little to no difference in terms of overall performance and could potentially lend some insight as to why individuals are inclined to trust cramming over spacing (Kornell 2009). If, at 24 h or weekly intervals, participants actually perform just as well having learned in one sitting, it is easy to understand why people might find this option more desirable when compared to spacing (Baddeley and Longman 1978; Son and Kornell 2009). It is also possible that the results of this study were underpowered and that is why there is no effect at shorter intervals.

The results of the second experiment show the beneficial effects of spaced learning for older adults when learning face-name associations, particularly at longer retention intervals. Although younger participants generally learned more and thus demonstrated better recall, older adults that had been spaced-trained were better able to retain the information that they had learned. Therefore, participants, irrespective of their age, who were spaced-trained performed significantly better than their massed-trained peers. These results are in accordance with the original hypotheses and the existing literature (Balota et al. 1989; Benjamin and Tullis 2010; Bercovitz et al. 2017). These other studies also suggest that older adults tend to perform poorly compared to younger adults, but they exhibit similar patterns of retention. Again, it is worth noting that the lack of findings at shorter intervals may be due to lack of power.

These findings could potentially be explained by encoding variability theory (Crowder 1976; Maddox 2016). Due to the delay between study intervals, spaced-trained participants have the opportunity to associate greater context with learned material, thus potentially making it easier to retrieve said material under various circumstances. Given that older adults are known to struggle when presented with context-specific cues, this could explain why younger adults perform better (Rabinowitz et al. 1982; Smith et al. 1998). However, encoding variability theory would suggest that older adults should not benefit from spacing at all, which is clearly not the case. Why, then, is spacing preserved with age? Findings of Callan and Schweighofer (2010) are consistent with deficient processing theory, the idea that spaced-trained individuals are more attentive to subsequent presentations when compared to massed-trained individuals. Due to the involvement of working memory at each stage in learning, massed-trained individuals are more inclined to believe themselves familiar with the material and therefore are less attentive on consecutive presentations. In contrast, spaced-trained participants are inclined to feel less familiar with the material which leads to more vigilant encoding with each presentation (Cepeda et al. 2006). If increased frontal activity is also present in older adults, this might explain the presence of the spacing effect, as well as aligning with other neuroimaging studies, for example, the posterior-anterior shift in ageing (PASA) model (Davis et al. 2007). Additionally, it is possible that due to over-activation or compensation-related brain activity, older adults are not able to distinguish between relevant and irrelevant contextual information the way young adults can, thus forming associations between target information and distractors (Campbell et al. 2010; Powell et al. 2018).

Furthermore, given that the face-name pairs task is thought to rely on the hippocampus (Smith et al. 2014), it is also possible that spaced advantages are due to the activation of this structure. For example, Li and Yang (2020) found that young spaced-trained participants showed significantly greater hippocampal activity when recognizing face-scene pairs compared to massed-trained participants. This activity was particularly pronounced at 1-month intervals. Given also the involvement of the hippocampus in consolidating long-term memories (Scoville and Milner 1957; Bercovitz et al. 2017; Delaney et al. 2018) and the importance of sleep in this process (Smolen et al. 2016), this could explain why distributed practice is so advantageous. It would be worth examining whether the same levels of hippocampal activation are observed in older adults.

In conclusion, these experiments have demonstrated that spaced learning is more advantageous than massed learning for both younger and older adults when attempting to retain face-name associations, particularly at longer intervals of 1 month. Furthermore, older adults perform significantly worse than younger adults under all conditions. However, spaced-trained individuals display similar patterns of forgetting at 1 month, regardless of age. Future studies may want to analyze face-name retention more specifically. The current studies only recorded correct versus incorrect responses. Analyses of specific face-name retention between trial 4 and the retest may shed further light on forgetting.

## Declarations

### Data availability statement

The data that support the findings of this study are available at: https://osf.io/x6esd/?view_only=fe1a5e36f8ce41bb81e6076041218a2f.
